# Serum Antibody and Glomerular Antigen of Antiphospholipase A2 Receptor in Chinese Patients with Idiopathic Membranous Nephropathy

**DOI:** 10.1155/2020/1693710

**Published:** 2020-05-08

**Authors:** Qiu-hua Zhang, Mian Wu, Zhi-gang Hu, Xiao-bin Liu, Biao Huang, Yi Zhang, Bin Liu, Yu-shan Zhu, Liang Wang, Zhu-xing Sun

**Affiliations:** ^1^Wuxi People's Hospital Affiliated to Nanjing Medical University, Wuxi 214000, China; ^2^Nanjing Medical University, Nanjing 211166, China; ^3^School of Life Science, Zhejiang, Sci-Tech University, Hangzhou 310018, China; ^4^Jiangsu Institute of Nuclear Medicine, Wuxi 214063, China

## Abstract

**Background:**

M-type phospholipase A2 receptor (PLA2R) is the first autoantigen responsible for idiopathic membranous nephropathy (IMN). However, serum PLA2R antibody (PLA2R-Ab) can be inaccurate in distinguishing between IMN and secondary membranous nephropathy, while renal PLA2R antigen (PLA2R-Ag) emerges as an ancillary diagnostic. The present study is aimed at examining the associations between PLA2R-Ab in sera and PLA2R-Ag in kidneys in IMN patients.

**Methods:**

A total of 93 patients with IMN were retrospectively identified. Their serum PLA2R-Ab and renal PLA2R-Ag expression levels were determined, and the clinical correlations between these parameters and clinical features were examined.

**Results:**

The sensitivities of serum PLA2R-Ab and renal PLA2R-Ag for diagnosing IMN were 74.2% and 88.2%, respectively (*P* < 0.001), with poor consistency. Higher serum PLA2R-Ab levels were correlated to stronger renal PLA2R-Ag expression (*P* = 0.048). Patients with positive PLA2R-Ab significantly differed from those with negative levels, in terms of proteinuric levels over 24 hours (4.54 *vs.* 3.46 g/day, *P* = 0.015) and serum albumin (23.28 *vs.* 27.95 g/L, *P* = 0.038). Among patients with positive renal PLA2R-Ag, patients with positive PLA2R-Ab had significantly higher 24-hour proteinuria, when compared to patients with negative PLA2R-Ab (4.57 *vs.* 3.08 g/day, *P* = 0.005). Among those with positive PLA2R-Ab in sera, their PLA2R-Ab levels were correlated with the estimated glomerular filtration and serum creatinine.

**Conclusion:**

Serum PLA2R-Ab exhibits a closer correlation with proteinuric severity and renal function, when compared to renal PLA2R-Ag.

## 1. Introduction

Membranous nephropathy (MN) has been reported to be the most common etiology of nephrotic syndrome in nondiabetic patients [1]. Among all patients with MN, nearly 80% can be attributed to idiopathic membranous nephropathy (IMN), while the remaining cases are secondary MN (SMN) related to diseases, including rheumatologic illnesses (systemic lupus erythematosus (SLE)), infections (hepatitis or syphilis), cancers, and medications [2, 3]. Antigens responsible for IMN have been extensively studied using both experimental and clinical approaches. The M-type phospholipase A2 receptor (PLA2R) is a podocytic transmembrane receptor and is the first identified autoantigen of IMN. Circulating PLA2R antibody (PLA2R-Ab) is detectable in approximately 70% of IMN patients [4]. A genome-wide association study suggested that PLA2R-Ab is involved in the development of IMN [5]. Upon initial discovery, the specificity of PLA2R-Ab for diagnosing IMN is 100% [4]. However, recent studies have shown that cases of SMN with positive PLA2R-Ab exist [6, 7]. In line with these findings, PLA2R-Ab may be an effective biomarker for differentiating between patients with IMN and patients with SMN.

On the other hand, renal PLA2R expression in biopsy specimens can also assist in the diagnosis, in addition to circulatory PLA2R-Ab. In normal or non-IMN glomerular diseased kidneys, renal PLA2R antigen (RLA2R-Ag) express weakly with a linear staining pattern in podocytes, while in kidneys obtained from IMN patients, strong granular staining is common, which is called “positive staining” [7, 8].

Patients with IMN and positive serum PLA2R-Ab often have positive PLA2R-Ag staining in glomeruli [9]. However, the associations between serum PLA2R-Ab and renal PLA2R-Ag in IMN patients are less well-characterized, necessitating more studies. Furthermore, the accuracy of methods for measuring serum PLA2R-Ab substantially varies among studies, rendering difficulties in result interpretation. The investigators previously utilized a time-resolved fluoroimmunoassay (TRFIA) with high sensitivity to quantify serum PLA2R-Ab in IMN patients [10–12]. In the present study, the relationship among serum PLA2R-Ab, renal PLA2R-Ag, and the clinical features of these patients were analyzed.

## 2. Materials and Methods

### 2.1. Participant Identification and Enrollment

A total of 93 patients with biopsy-proven IMN from the Affiliated Wuxi People's Hospital of Nanjing Medical University in China were retrospectively identified between 2013 and 2017. Patients with SMN, including those related to systemic autoimmune diseases (lupus nephritis, Sjogren's syndrome, and others), infections (syphilis, human immunodeficiency virus, and hepatitis B or C), and malignancies, or those related to toxin exposure, were excluded. Sera and urine were collected, and renal biopsy was performed during the blood sampling. None of these patients received immunosuppression prior to study enrollment, and their medications were continued for hypertension and proteinuria.

The study procedure was approved by the Ethics Committee of Affiliated Wuxi People's Hospital of Nanjing Medical University (No. Kyl2016001) and adhered to the 2008 Declaration of Helsinki and good clinical practice guidelines.

### 2.2. Sample Preparation and Assessment

Serum creatinine and urea were measured, and the estimated glomerular filtration rate (eGFR) was calculated based on the chronic kidney disease epidemiology collaboration (CKD-EPI) equation [13]. Urine was measured using the average of three 24-hour samples.

### 2.3. Examination of Renal Pathology and the Immunostaining of PLA2R Antigen

Light microscopy, immunofluorescence (IF), and electron microscopy were used to examine the specimens obtained from the renal biopsy. Direct IF with regard to immunoglobulin G (IgG), IgA, IgM, C3, C4, and C1q was also performed on the frozen sections, with the presentation of semiquantitative results (0 to 4+). Dense deposits were semiquantitatively evaluated (0 to 3+) based on the findings obtained from the electron microscopy.

Renal PLA2R staining was performed in the paraffin-embedded sections using polyclonal anti-PLA2R1 antibodies from rabbits (Sigma, USA) at 1 : 500 dilution after 100% power microwaving for 18 minutes, followed by donkey anti-rabbit FITC IgG (Millipore, USA) treatment at 1 : 100 dilution. The staining intensity was evaluated by immunofluorescence microscopy (Nikon ECLIPSE 80i, Japan), and the positive PLA2R staining was defined based on the granularity along the capillary loops (0 to 3+). Negative controls (secondary antibody only) were used in all experiments to rule out the possibility of cross-reactivity between the human IgG and secondary antibody.

### 2.4. Serum PLA2R-Ab Detection Using Time-Resolved Fluoroimmunoassay (TRFIA)

The investigators made their own kits to detect the serum PLA2R-Ab using TRFIA. An AutoDELFIA1235 automatic immunoassay system from Perkin Elmer was used to measure the Eu3+ fluorescence in microtiter wells. Recombinant PLA2R (rPLA2R) was coated onto 96-well plates to capture the PLA2R antibody, and goat-anti-human IgG tracers with europium chelate were prepared to detect the presence of the antigen. After washing, the fluorescence counts of the bound tracer were measured, and the serum anti-PLA2R-IgG was quantified. A purified anti-PLA2R-IgG calibrator was also used to ensure result consistency. The detection range for anti-PLA2R-IgG based on TRFIA was 0.02-340.00 mg/L, with an intra- and interassay coefficient of variation of 3.2% and 5.6%, respectively. Serum PLA2R − Ab > 0.91 mg/L was defined as positive [10–12].

### 2.5. Statistical Analysis

The statistical analysis was conducted using SPSS (version 18; SPSS, Inc., Chicago, Ill., USA). Continuous data were presented as mean ± standard error (SE), and *t*-test was used for parametric analyses. Categorical variables were described as frequencies or percentages and were analyzed by Chi-square test or Fisher's exact test. Furthermore, the correlation among parameters was analyzed using one-way analysis of variance (ANOVA). In addition, Cohen's Kappa was used to analyze the agreement between renal PLA2R-Ag and serum PLA2R-Ab. A *P* value of <0.05 was considered statistically significant.

## 3. Results

### 3.1. Patterns of Distribution for Serum PLA2R-Ab and Renal PLA2R-Ag

**Figure 1:**
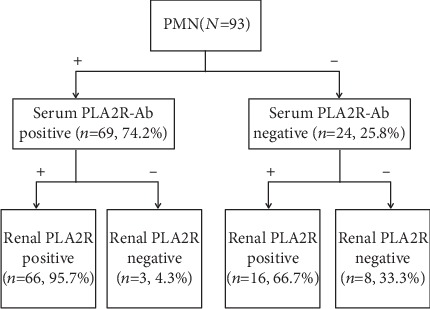
The pattern of distribution for serum PLA2R-Ab and renal PLA2R-Ag among participants; -, negative; +, positive.

**Figure 2:**
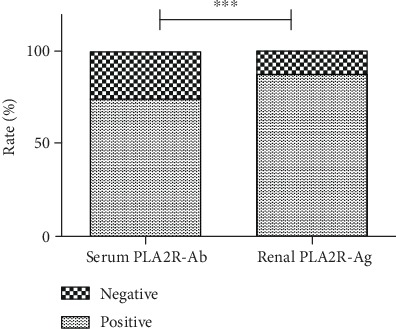
The proportion of patients with positive serum PLA2R-Ab and positive renal PLA2R-Ag. Chi-square test was used to compare the positivity rates; ^∗∗∗^*P* < 0.001.

In the present study, 74.2% of patients (69/93) were positive for serum PLA2R-Ab, while 88.2% of patients (82/93) were positive for renal PLA2R-Ag (*P* = 0.001, Figure 1). Among the 93 patients, 66 patients had positive serum PLA2R-Ab and renal PLA2R-Ag at the same time, while three and 16 patients had serum-positive/kidney-negative and serum-negative/kidney-positive, respectively (Figure 1). Furthermore, negative serum and kidney assays were noted for eight patients. It was calculated that the sensitivity for serum PLA2R-Ab and renal PLA2R-Ag in IMN was 74.2% and 88.2%, respectively (*P* < 0.001, Figure 2).

### 3.2. Consistency between Renal PLA2R-Ag and Serum PLA2R-Ab for IMN

**Figure 3:**
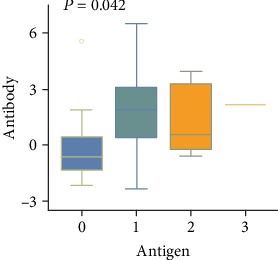
The correlation between renal PLA2R-Ag and serum PLA2R-Ab. The serum PLA2R-Ab levels were log10 transformed.

Among the 69 patients with positive serum PLA2R-Ab, 66 patients also had positive renal PLA2R-Ag (95.7%), while among the 24 patients with negative serum PLA2R-Ab, 16 (66.7%) patients had positive renal PLA2R-Ag (Figure 1). The Kappa value was 0.352, suggesting that the consistency for diagnosing IMN between PLA2R-Ab in sera and PLA2R-Ag in kidneys was poor. There was a statistical difference of serum PLA2R-Ab in different kidney PLA2R-Ag groups (*P* = 0.042, Figure 3). In the group with a strong expression of PLA2R-Ag in kidneys, the level of serum antibody was also high. These above findings indicate that the increase in serum PLA2R-Ab levels correlated to the stronger expression of renal PLA2R-Ag.

### 3.3. The Relationship between PLA2R-Ab in Sera or PLA2R-Ag in Kidneys and Other Clinical Biomarkers

Patients with positive PLA2R-Ab significantly differed from those with negative levels, in terms of 24-hour urine protein (4.54 *vs.* 3.46 g/day, *P* = 0.015) and serum albumin (23.28 *vs.* 27.95 g/L, *P* = 0.038) (Table 1). However, no differences were observed between patients with positive and negative renal PLA2R-Ag, in terms of 24-hour urine protein, serum albumin, creatinine, urea, and eGFR.

Among patients with positive renal PLA2R-Ag, patients with positive PLA2R-Ab significantly differed from those with negative levels, in terms of 24-hour urine protein (4.57 *vs.* 3.08 g/day, *P* = 0.005; Table 2). However, among patients with positive serum PLA2R-Ab, no difference was observed between patients with positive and negative renal PLA2R-Ag, in terms of 24-hour urine protein, serum albumin, creatinine, urea, and eGFR.

**Figure 4:**
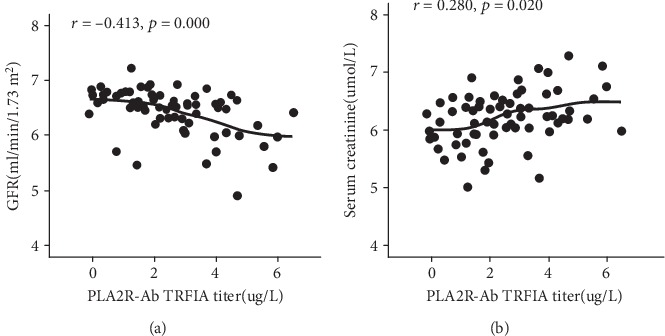
The correlations between serum PLA2R-Ab and clinical biomarkers, including eGFR and serum creatinine. The serum PLA2R-Ab titers negatively correlated with eGFR (a), but positively correlated with serum creatinine (b). Serum PLA2R-Ab, eGFR, and creatinine were log2 transformed.

The correlations between PLA2R-Ab in sera or PLA2R-Ag in kidneys and clinical biomarkers among patients with positive serum PLA2R-Ab were also analyzed. The serum PLA2R-Ab level was significantly correlated with serum creatinine and eGFR, but not with serum albumin, urea, or 24-hour urine protein (Table 3). The serum PLA2R-Ab titers were negatively correlated with eGFR, but were positively correlated with serum creatinine (Figure 4). However, among patients with positive renal PLA2R-Ag, no significant correlations were found between the renal PLA2R-Ag expression and 24-hour urine protein, serum albumin, creatinine, urea, and eGFR.

## 4. Discussion

In the present study, serum PLA2R-Ab was detectable in 74.2% of patients with IMN using TRFIA, while renal PLA2R-Ag was detectable in 88.2% of patients using IF. The prevalence of serum PLA2R-Ab in other studies in literature varied between 57% and 82% [14–20]. A recent meta-analysis that addressed the overall diagnostic ability of PLA2R-Ab in sera and PLA2R antigen in kidneys for IMN revealed that the pooled sensitivity of serum PLA2R-Ab and renal PLA2R-Ag for identifying IMN was 65% and 79%, respectively [21]. On the other hand, a higher sensitivity of PLA2R-Ab in sera for recognizing IMN was found [17, 21]. The previous study conducted by the investigators suggested that the sensitivity and range of detection for PLA2R-Ab using TRFIA were better and wider, when compared to those that used enzyme-linked immunosorbent assay (ELISA) [11]. In addition, the present study also revealed the higher sensitivity of renal PLA2R-Ag for IMN in a Chinese cohort, which is consistent with the findings of other studies [9, 22].

In order to compare the performance of PLA2R-Ab in sera and PLA2R-Ag in kidneys, the relationship between PLA2R-Ab in sera and PLA2R-Ag in kidneys was only assessed in incident IMN patients, whose sera were collected at the time of renal biopsy without prior immunosuppression. It was observed that the consistency between serum PLA2R-Ab and renal PLA2R-Ag in IMN patients was poor, but high serum PLA2R-Ab levels were correlated with stronger renal PLA2R-Ag expression. It was also found that three patients had positive serum PLA2R-Ab, but had negative renal PLA2R-Ag. Hence, the investigators propose that renal PLA2R-Ag can be falsely negative in patients with IMN, because antigen-antibody depositions can be cleared with antigenicity disappearance. For the 16 patients with negative serum PLA2R-Ab, but with positive renal PLA2R-Ag, the investigators also propose that this discrepancy may have been caused by the rapid clearance of PLA2R-Ab, or the delayed serum sampling and disappearance of the preformed antibody. Under this condition, the persistent proteinuria can result from the irreversible ultrastructural changes in podocytes [20, 23]. Consequently, the combined measurement of serum PLA2R-Ab and renal PLA2R-Ag may improve the overall performance of PLA2R for IMN. However, this finding requires further exploration.

Beck and Salant speculated that the negative serum PLA2R-Ab resulted from the cessation of immunologic activity at the time of serum sampling [24]. Similarly, other researchers suggest that serum PLA2R-Ab is a surrogate of immunologic activity and predicts clinical outcomes [1]. In the present study, 24-hour urine protein was significantly higher and serum albumin was significantly lower in patients with positive PLA2R-Ab, when compared to patients with negative levels, which is consistent with a previous report [12]. This might represent the higher clinical severity of a subpopulation with positive PLA2R-Ab. However, there was no significant difference between patients with positive and negative renal PLA2R-Ag. The clinical differences between patients with positive and negative serum PLA2R-Ab among those with positive renal PLA2R-Ag were further compared, and it was discovered that significant differences in proteinuria existed, while there were no significant differences in serum albumin levels. However, the serum albumin levels were lower in patients with positive serum PLA2R-Ab than in patients with negative serum PLA2R-Ab. This is a phenomenon attributable to the modest sample size. Furthermore, there was no significant difference between patients with positive and negative renal PLA2R-Ag among patients with positive serum PLA2R-Ab. The investigators consider that serum PLA2R-Ab may be more closely correlated with disease activity than renal PLA2R-Ag in patients with IMN.

It was also found that serum PLA2R-Ab levels are correlated with serum creatinine and eGFR in patients with positive serum PLA2R-Ab, but such a correlation was not found in patients with positive renal PLA2R-Ag. The investigators consider that serum PLA2R-Ab may be more closely correlated with renal function, when compared to renal PLA2R-Ag, in patients with IMN.

The present study had several limitations. The present study had a retrospective study design and a small sample size. In addition, although the total IgG of serum PLA2R was tested, the type of serum anti-PLA2R antibody varied among patients. IgG4 was the main antibody subtype in IMN patients, while IgG1 and IgG2 were the main subtypes in SMN patients [25]. Further studies are needed to determine the importance of anti-PLA2R antibody subtypes in MN. In addition, it has been found that Thrombospondin Type-1 Domain–Containing 7A (THSD7A) existed in IMN patients with negative PLA2R [26], which accounts for approximately 1%–5% of primary MN [27]. One study has identified that Exostosin 1 and Exostosin 2 are associated with PLA2R and THSD7A double-negative MN [27]. Therefore, further study on IMN-related antigens is needed. Nonetheless, the present study still has its importance.

## 5. Conclusion

In conclusion, serum PLA2R-Ab may be more closely correlated with disease activity and renal function, when compared to renal PLA2R-Ag. A combined measurement of serum PLA2R-Ab and renal PLA2R-Ag is expected to improve the overall performance for diagnosing IMN.

## Figures and Tables

**Table 1:** Clinical features of patients with positive and negative serum PLA2R-Ab.

Clinical features	Positive PLA2R-Ab (*n* = 69)	Negative PLA2R-Ab (*n* = 24)	*P* value
Serum creatinine (*μ*mol/L)	77.97 ± 24.30	93.77 ± 72.33	0.304
Serum urea (mmol/L)	4.74 ± 1.63	4.86 ± 3.03	0.855
eGFR (mL/min/1.73 m^2^)	90.03 ± 22.24	91.90 ± 28.66	0.743
Serum albumin (g/L)	23.28 ± 9.31	27.95 ± 9.53	0.038
24-hour urine protein (g/d)	4.54 ± 1.84	3.46 ± 1.80	0.015

**Table 2:** Differences in laboratory data among patients with positive renal PLA2R-Ag and between patients with positive and negative serum PLA2R-Ab.

Clinical features	Positive PLA2R-Ab (*n* = 66)	Negative PLA2R-Ab (*n* = 16)	*P* value
Serum creatinine (*μ*mol/L)	77.23 ± 24.21	108.56 ± 84.90	0.164
Serum urea (mmol/L)	4.69 ± 1.49	5.33 ± 3.40	0.474
eGFR (mL/min/1.73 m^2^)	90.98 ± 21.48	85.47 ± 32.43	0.410
Serum albumin (g/L)	23.10 ± 9.25	26.54 ± 8.77	0.182
24-hour urine protein (g/d)	4.57 ± 1.87	3.08 ± 1.78	0.005

**Table 3:** Correlations between serum PLA2R-Ab and clinical biomarkers in patients with positive serum PLA2R-Ab (*n* = 69).

Clinical features	Pearson correlation coefficient	*P* value
Serum creatinine (*μ*mol/L)	0.280	0.020
Serum urea (mmol/L)	0.087	0.479
eGFR (mL/min/1.73 m^2^)	-0.413	0.000
Serum albumin (g/L)	-0.030	0.805
24-hour urine protein (g/d)	-0.208	0.819

## Data Availability

The datasets generated and analyzed during the present study are available from the corresponding author on reasonable request.
